# Predictive factors of rapid linear renal progression and mortality in patients with chronic kidney disease

**DOI:** 10.1186/s12882-020-01982-8

**Published:** 2020-08-14

**Authors:** Ibrahim Ali, Rajkumar Chinnadurai, Sara T. Ibrahim, Darren Green, Philip A. Kalra

**Affiliations:** 1 Department of Renal Medicine, Salford Royal NHS Foundation Trust, Stott Lane, Salford, M6 8HD UK; 2 Department of Internal Medicine and Nephrology, Faculty of Medicine, Alexandria University, Alexandria, Egypt

**Keywords:** Chronic kidney disease, CKD, Progression, Prediction, Mortality, End-stage renal disease

## Abstract

**Background:**

Risk factors predictive of rapid linear chronic kidney disease (CKD) progression and its associations with end-stage renal disease (ESRD) and mortality requires further exploration, particularly as patients with linear estimated glomerular filtration rate (eGFR) trajectory represent a clear paradigm for understanding true CKD progression.

**Methods:**

A linear regression slope was applied to all outpatient eGFR values for patients in the Salford Kidney Study who had ≥2 years follow-up, ≥4 eGFR values and baseline CKD stages 3a-4. An eGFR slope (ΔeGFR) of ≤ − 4 ml/min/1.73m^2^/yr defined rapid progressors, whereas − 0.5 to + 0.5 ml/min/1.73m^2^/yr defined stable patients. Binary logistic regression was utilised to explore variables associated with rapid progression and Cox proportional hazards model to determine predictors for mortality prior to ESRD.

**Results:**

There were 157 rapid progressors (median ΔeGFR − 5.93 ml/min/1.73m^2^/yr) and 179 stable patients (median ΔeGFR − 0.03 ml/min/1.73m^2^/yr). Over 5 years, rapid progressors had an annual rate of mortality or ESRD of 47 per 100 patients compared with 6 per 100 stable patients. Factors associated with rapid progression included younger age, female gender, higher diastolic pressure, higher total cholesterol:high density lipoprotein ratio, lower albumin, lower haemoglobin and a urine protein:creatinine ratio of > 50 g/mol. The latter three factors were also predictive of mortality prior to ESRD, along with older age, smoking, peripheral vascular disease and heart failure.

**Conclusions:**

There is a heterogenous interplay of risk factors associated with rapid linear CKD progression and mortality in patients with CKD. Furthermore, rapid progressors have high rates of adverse outcomes and require close specialist monitoring.

## Background

Chronic kidney disease (CKD) is an important public health concern given that lower estimated glomerular filtration rate (eGFR) and increasing albuminuria are common and are independent risk factors associated with progression to end-stage renal disease (ESRD), cardiovascular events and all-cause mortality [1].

Accurately stratifying patients with CKD who are at risk of progression could enable earlier, targeted treatment in an effort to stabilise renal decline and reduce future adverse outcomes [2]. Data from epidemiological studies have been used to create risk calculators for the prediction of outcomes such as ESRD and mortality in patients with CKD [3, 4]. However, they have yet to be implemented in routine clinical practice and require further refinement [5]. One particular omission from current prediction tools involves quantifying the rate of change in renal function in patients over time, which can help conceptualise an individual’s risk profile more meaningfully [6, 7]. Although a number of studies have explored the association of various risk factors on different rates of progression [8–10], there is a lack of data focusing exclusively on patients with a consistent linear rate of progression and the associations with adverse outcomes such as ESRD and mortality. These patients warrant attention as their linear eGFR trajectory represents a clear paradigm for understanding true CKD progression.

In this study we focus on patients with a linear pattern of progression stratified into two groups – rapid progressors or stable patients – defined by their rate of eGFR change. We aimed to (1) determine factors predictive of rapid linear CKD progression; (2) evaluate whether these factors are different depending upon the underlying disease aetiology; (3) determine the variables associated with mortality prior to ESRD in rapid progressors and stable patients and (4) explore how the rate of the eGFR trajectory impacts on outcomes of ESRD and mortality.

## Methods

### Patient population

The Salford Kidney Study (SKS) is a prospective observational cohort study based in the United Kingdom that has been recruiting patients with non-dialysis dependent CKD since 2002. Any patient referred to the renal services at Salford Royal NHS Foundation Trust who is ≥18 years old with an eGFR of < 60 ml/min/1.73m^2^ is eligible for recruitment.

### Baseline covariates

All covariates were measured at the point of recruitment into SKS. Demographic data in this analysis included age, gender, ethnicity, history of current or past smoking, body mass index (BMI), systolic (SBP) and diastolic blood pressure (DBP). Co-morbidities included hypertension, diabetes mellitus (DM), myocardial infarction (MI), peripheral vascular disease (PVD), stroke and heart failure (HF). Medications of interest included use of angiotensin converting enzyme inhibitor (ACEi), angiotensin receptor blockers (ARB) and statins. Laboratory values included serum creatinine, eGFR calculated using the CKD-EPI equation, bicarbonate, urea, calcium, phosphate, alkaline phosphatase, albumin, total cholesterol:high density lipoprotein (HDL) ratio, C-reactive protein, haemoglobin (Hb) and urine protein:creatinine ratio (uPCR), where uPCR values of < 15 g/mol, 15-50 g/mol and > 50 g/mol categorised patients into albuminuria grades of A1, A2 and A3 respectively, based on international guidelines [11]. Subsequent blood tests performed at routine clinic visits were accessible via the hospital’s electronic patient record and were used to define a patient’s rate of progression.

### Inclusion criteria and study outcomes

**Fig. 1:**
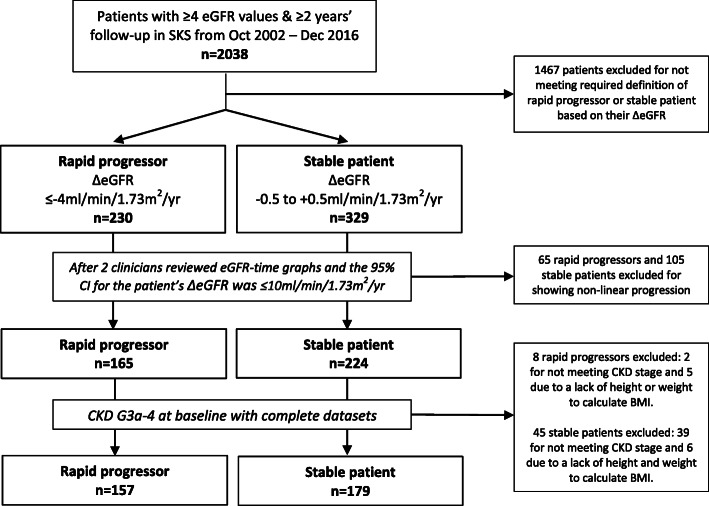
Patient selection from the Salford Kidney Study (SKS)

Patient selection into this study was performed retrospectively and involved 2 stages (Fig. 1). First, linear regression was applied to all outpatient eGFR values for patients with at least 4 eGFR measurements and 2 years follow-up [12, 13] in order to obtain a delta (Δ) eGFR slope (ml/min/1.73m^2^/yr). The outpatient eGFR values used to calculate the ΔeGFR for each patient represent all the tests performed in clinic as part of a patient’s renal follow-up. Rapid progression was defined as a ΔeGFR of ≤ − 4 ml/min/1.73m^2^/yr (i.e. losing more than 4 ml/min/1.73m^2^/yr) [10, 14]. Stable patients were defined as a ΔeGFR of − 0.5 to + 0.5 ml/min/1.73m^2^/yr as this small range centred on a zero rate of change. Second, visual inspection of the eGFR-time graphs, a methodology that has been used previously [15], helped to corroborate the linear pattern of progression, and patients with non-linear progression were excluded. This phase was performed by two clinicians independently as a means to ensure reproducibility. We also calculated the 95% confidence intervals (CI) for the ΔeGFR of each patient. Those with a smaller size interval are by definition expected to have a more consistent linear pattern than those with larger intervals. We therefore set a cut-off 95% CI of ≤10 ml/min/1.73m^2^/yr for each patient as a quantitative marker of eGFR linearity. Finally, only patients with baseline CKD G3a-4 (eGFR 15 to < 60 ml/min/1.73m^2^) comprised the final cohort. Patient data was reviewed until 31st December 2019 for study outcomes including reaching ESRD or death prior to ESRD. ESRD was defined as initiation of chronic haemodialysis or peritoneal dialysis, receiving a renal transplant or initiating follow-up in the conservative care clinic.

### Statistical analysis

Continuous data is presented as median ± interquartile range; categorical data as number (percentage). To compare variables between rapid progressors and stable patients, Mann-Whitney U or chi-squared test were used for continuous and categorical variables respectively. Binary logistic regression modelling was used to determine predictors associated with rapid CKD progression across all patients and in three specific conditions: diabetic nephropathy, glomerulonephritis of any cause and hypertensive nephropathy. These conditions were selected as patient numbers permitted appropriate analysis. Cox proportional hazards ratios with 95% CIs were calculated to determine factors implicated in mortality prior to ESRD in both rapid progressors and stable patients. The assumption of proportional hazards was assessed by the non-significance of each time-by-variable interaction (an interaction between a variable and a linear function of time) in both patient groups (Additional file 1: Tables S1 and S2). Kaplan-Meier survival curves for ESRD and mortality prior to ESRD used Log Rank significance testing. To account for competing risks, the competing event was censored in survival analyses [16]. All multivariate models used a forward stepwise elimination procedure [17] incorporating the following 22 baseline clinical variables: age, gender, SBP, DBP, BMI, hypertension, DM, smoking, MI, PVD, stroke, HF, ACEi/ARB use, statin use, eGFR, bicarbonate, calcium, phosphate, albumin, Hb, total cholesterol:HDL ratio and A3 proteinuria. Statistical significance in all analyses was defined as *p* < 0.05. Analyses were undertaken using SPSS (Version 25.0) (IBM SPSS, Chicago, IL) licensed to the University of Manchester.

## Results

### Baseline characteristics

A total of 157 patients with rapid linear progression and 179 stable patients comprised the final cohort (Table 1). There was no disagreement between the two clinicians during visual inspection of the eGFR-time graphs with respect to selecting patients with linear progression. Quantitatively, eGFR linearity was reflected in the average 95% CI of the ΔeGFR for rapid progressors of only 2.0 ml/min/1.73m^2^/yr and 1.7 ml/min/1.73m^2^/yr in stable patients.

**Table 1:** Baseline characteristics of rapid progressors and stable patients

Variable	Rapid progressor (*n* = 157)	Stable patient (*n* = 179)	*P*-value
Age (years)	54.0 (43.5–64.0)	68.4 (58.8–76.5)	**< 0.001**
Men, *n* (%)	81 (52)	128 (72)	**< 0.001**
Caucasian, *n* (%)	152 (97)	174 (97)	0.833
Systolic blood pressure (mmHg)	144 (133–157)	137 (122–148)	**0.001**
Diastolic blood pressure (mmHg)	82 (74–91)	74 (66–80)	**< 0.001**
Hypertension, *n* (%)	151 (96)	168 (94)	0.332
Diabetes, *n* (%)	41 (26)	67 (37)	**0.027**
Body mass index (kg/m^2^)	28.0 (24.5–32.0)	28.0 (24.5–32.2)	0.925
Past/current smoking history, *n* (%)	100 (64)	122 (68)	0.389
Myocardial infarction, *n* (%)	5 (3)	25 (14)	**0.002**
Peripheral vascular disease, *n* (%)	8 (5)	11 (6)	0.375
Stroke, *n* (%)	11 (7)	5 (3)	0.138
Heart failure, *n* (%)	2 (1)	10 (6)	**0.047**
ACEi/ARB, *n* (%)	112 (71)	118 (66)	0.286
Statin, *n* (%)	92 (58)	116 (65)	0.243
CKD stage 3, *n* (%)	109 (69)	78 (44)	**< 0.001**
CKD stage 4, *n* (%)	48 (31)	101 (56)	**< 0.001**
Years follow-up	3.9 (2.9–5.0)	7.5 (5.7–9.8)	**< 0.001**
**Primary renal disease**			
Diabetic nephropathy, *n* (%)	31 (20)	39 (22)	0.646
ADPKD, *n* (%)	52 (33)	2 (1)	**< 0.001**
Hypertensive nephropathy, *n* (%)	11 (7)	17 (10)	0.410
Renovascular disease, *n* (%)	3 (2)	14 (8)	**0.014**
Obstructive uropathy, *n* (%)	7 (4)	17 (9)	**0.038**
Glomerulonephritis, *n* (%)	26 (17)	24 (13)	0.418
Other causes, *n* (%)	21 (13)	39 (22)	**0.045**
Unknown, *n* (%)	6 (4)	27 (15)	**< 0.001**
**Laboratory results**			
Creatinine (umol/l)	171 (145–201)	193 (157–238)	**< 0.001**
eGFR-EPI (ml/min/1.73m^2^)	34 (28–41)	28 (22–37)	**< 0.001**
eGFR measurements, *n*	25 (16–36)	24 (15–38)	0.960
Days between eGFR measurements, *n*	47 (24–91)	84 (39–135)	**< 0.001**
ΔGFR (±ml/min/1.73m^2^/yr)	−5.930 (−7.345 to −4.810)	−0.030 (−0.290 to 0.170)	**< 0.001**
Bicarbonate (mmol/L)	22.5 (20.2–25.0)	23.0 (20.7–24.9)	0.354
Urea (mmol/L)	12.0 (9.6–15.0)	13.4 (10.8–17.6)	**0.001**
Calcium (mmol/L)	2.31 (2.21–2.37)	2.28 (2.21–2.37)	0.350
Phosphate (mmol/L)	1.16 (1.03–1.29)	1.05 (0.93–1.21)	**< 0.001**
Alkaline phosphatase (mmol/L)	78 (59–95)	83 (65–104)	**0.025**
Albumin (g/L)	41 (38–44)	44 (42–46)	**< 0.001**
Total cholesterol/HDL ratio	3.55 (2.75–4.46)	3.17 (2.48–4.06)	**0.007**
C-reactive protein (mg/L)	2.8 (1.2–7.3)	2.5 (1.0–5.7)	0.234
Haemoglobin (g/L)	122 (113–134)	129 (119–137)	**0.006**
Urine protein:creatinine ratio (g/mol)	102 (28–289)	17 (9–36)	**< 0.001**
- A1 proteinuria (< 15 g/mol)	16 (10)	76 (42)	**< 0.001**
- A2 proteinuria (15-50 g/mol)	44 (26)	73 (41)	**0.005**
- A3 proteinuria (> 50 g/mol)	107 (64)	30 (17)	**< 0.001**

Continuous data are presented as median (interquartile range) and categorical variables presented as number (percentage)

*P*-value calculated by Mann-Whitney test for continuous data and Chi-squared test for categorical data

Abbreviations: *ADPKD* (Autosomal dominant polycystic kidney disease); *ACEi* (Angiotensin-converting enzyme inhibitor); *ARB* (Angiotensin receptor blocker); *eGFR-EPI* (eGFR calculated using the CKD-EPI equation)

The two patient groups demonstrated a clear separation in ΔeGFR: rapid patients progressed at a median rate of − 5.93 ml/min/1.73m^2^/yr (with the median upper and lower 95% CIs of − 5.41 to − 7.42), whereas the eGFR changed at a rate of only − 0.03 ml/min/1.73m^2^/yr (median 95% CIs 0.81 to − 0.89) in stable patients (*p* < 0.001). This was despite the baseline eGFR being lower in the stable group (28 ml/min/1.73m^2^ versus 34 ml/min/1.73m^2^; *p* < 0.001). Each patient group had the same large number of eGFR measurements per patient (median of 25), with the frequency of monitoring higher for rapid progressors: median of 47 (24–91) days between eGFR testing in contrast to 84 days (38–135) in stable patients; *p* < 0.001. The median follow-up time for the whole cohort was 5.3 years but rapid progressors had a much shorter follow-up of 3.9 years compared with 7.5 years in stable patients.

There was a significantly higher proportion of younger, female patients with higher blood pressure amongst the rapid progressors. In contrast, stable patients had a higher proportion with cardiovascular co-morbidity, including a history of MI and HF. There was no difference between the groups with respect to ACEi, ARB or statin use. Autosomal dominant polycystic kidney disease (ADPKD) was the commonest primary renal disease in rapid progressors, accounting for 33% of cases in this group, whereas there were more patients with renovascular disease or obstructive nephropathy in the stable group. Rapid progressors also had markedly higher levels of proteinuria and this was reflected in the majority of patients being categorised with A3 proteinuria.

### Factors associated with rapid linear CKD progression

Univariate analysis of the factors associated with rapid linear progression are presented in Additional file 1: Table S3. In multivariate analysis, younger age, female gender, higher DBP, lower albumin, higher total cholesterol:HDL ratio, lower Hb and A3 proteinuria were all independently associated with rapid progression (Table 2). A3 proteinuria imparted the highest adjusted odds ratio (OR) of being a rapid progressor: 7.66, 95% CI 3.77–15.56, *p* < 0.001.

**Table 2:** Predictors of rapid linear progression based on binary logistic regression modelling

Variable	Adjusted OR	95% CI	*P*-value
Age (per year)	0.958	0.936–0.980	< 0.001
Male	0.300	0.154–0.585	0.002
DBP (per 1 mmHg)	1.063	1.033–1.093	< 0.001
Total cholesterol:HDL ratio	1.346	1.047–1.730	0.020
Albumin (per 1 g/L)	0.912	0.842–0.987	0.023
Hb (per 1 g/L)	0.956	0.935–0.979	0.004
A3 proteinuria	7.661	3.772–15.560	< 0.001

Abbreviations: *DBP* (Diastolic blood pressure); *HDL* (High density lipoprotein); *Hb* (Haemoglobin)

### Factors associated with progression in specific conditions

The baseline characteristics of patients with diabetic nephropathy, glomerulonephritis of any cause and hypertensive nephropathy are provided in Additional file 1: Table S4. Different combinations of clinical factors were associated with rapid progression in these specific conditions (Table 3).

**Table 3:** Predictors of rapid linear progression based on binary logistic regression modelling in different causes of CKD

Variable	Diabetic nephropathy			Glomerulonephritis			Hypertensive nephropathy		
	OR	95% CI	*P*-value	OR	95% CI	*P*-value	OR	95% CI	*P*-value
Age (per year)							1.055	1.007–1.105	0.023
A3 proteinuria	13.393	4.510–39.771	< 0.001	26.120	5.253–129.864	< 0.001	11.530	2.335–56.930	0.003
Albumin (per 1 g/L)				0.888	0.817–0.965	0.005			
Hb (per 1 g/L)	0.958	0.933–0.984	0.002						
Body mass index (per 1 kg/m^2^)				1.120	1.036–1.212	0.001			

Abbreviations: *Hb* (Haemoglobin)

A3 proteinuria conferred the highest adjusted OR across all the diseases but differentiating factors for rapid progression included lower Hb in diabetic nephropathy (OR 0.96, 95% CI 0.93–0.98, *p* = 0.002), lower albumin in glomerulonephritis (OR 0.89, 95% CI 0.82–0.97, *p* = 0.005), and older age in hypertensive nephropathy (OR 1.06, 95% CI 1.01–1.11, *p* = 0.023).

### Factors associated with mortality in rapid linear progressors and stable patients

Univariate analyses of the clinical factors associated with mortality in rapid progressors and stable patients are presented in Additional file 1: Tables S5 and S6. In multivariate analysis, older age, male gender, a lack of ACEi/ARB blockade, MI, acidosis and anaemia were significantly associated with mortality prior to ESRD in rapid progressors. Older age and anaemia were also contributory in stable patients but smoking, PVD, HF and A3 proteinuria were specifically relevant in this patient cohort (Table 4).

**Table 4:** Cox proportional hazards ratio for predictive factors for mortality prior to ESRD

	IN RAPID PROGRESSOR			IN STABLE PATIENT		
Variable	HR	95% CI	*P*-value	HR	95% CI	*P*-value
Age (per year)	1.176	1.117–1.238	< 0.001	1.091	1.061–1.121	< 0.001
Male	3.501	1.382–8.867	0.008			
Smoking				1.834	1.015–3.314	0.045
ACEi/ARB	0.222	0.081–0.610	0.004			
MI	3.711	1.739–7.918	0.001			
PVD				2.014	1.173–3.458	0.011
HF				2.423	1.468–4.000	0.001
Bicarbonate (per mmol/L)	0.838	0.717–0.979	0.026			
Hb (per 1 g/L)	0.918	0.885–0.952	< 0.001	0.964	0.947–0.981	< 0.001
A3 proteinuria				2.554	1.333–4.894	0.005

Abbreviations: *MI* (Myocardial infarction); *PVD* (Peripheral vascular disease); *HF* (Heart failure); *Hb* (Haemoglobin)

### Impact of ΔeGFR on ESRD and mortality

**Fig. 2:**
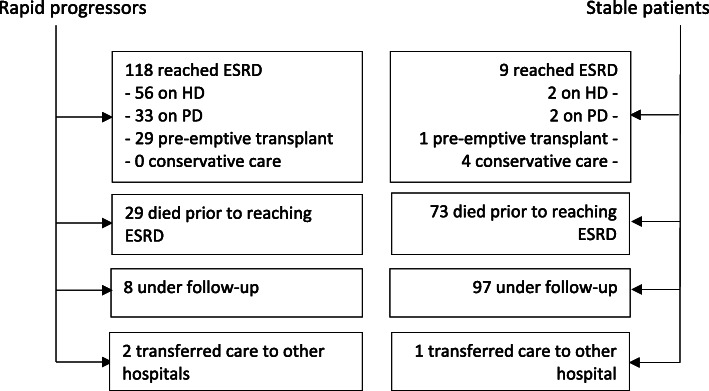
Outcomes for rapid progressors and stable patients. Abbreviations: HD (haemodialysis); PD (peritoneal dialysis)

Over a cumulative follow-up of 2366 patient-years in the combined cohort of rapid progressors and stable patients, 127 patients reached ESRD, 102 died prior to ESRD and 105 remained under nephrology follow-up (Fig. 2).

**Fig. 3:**
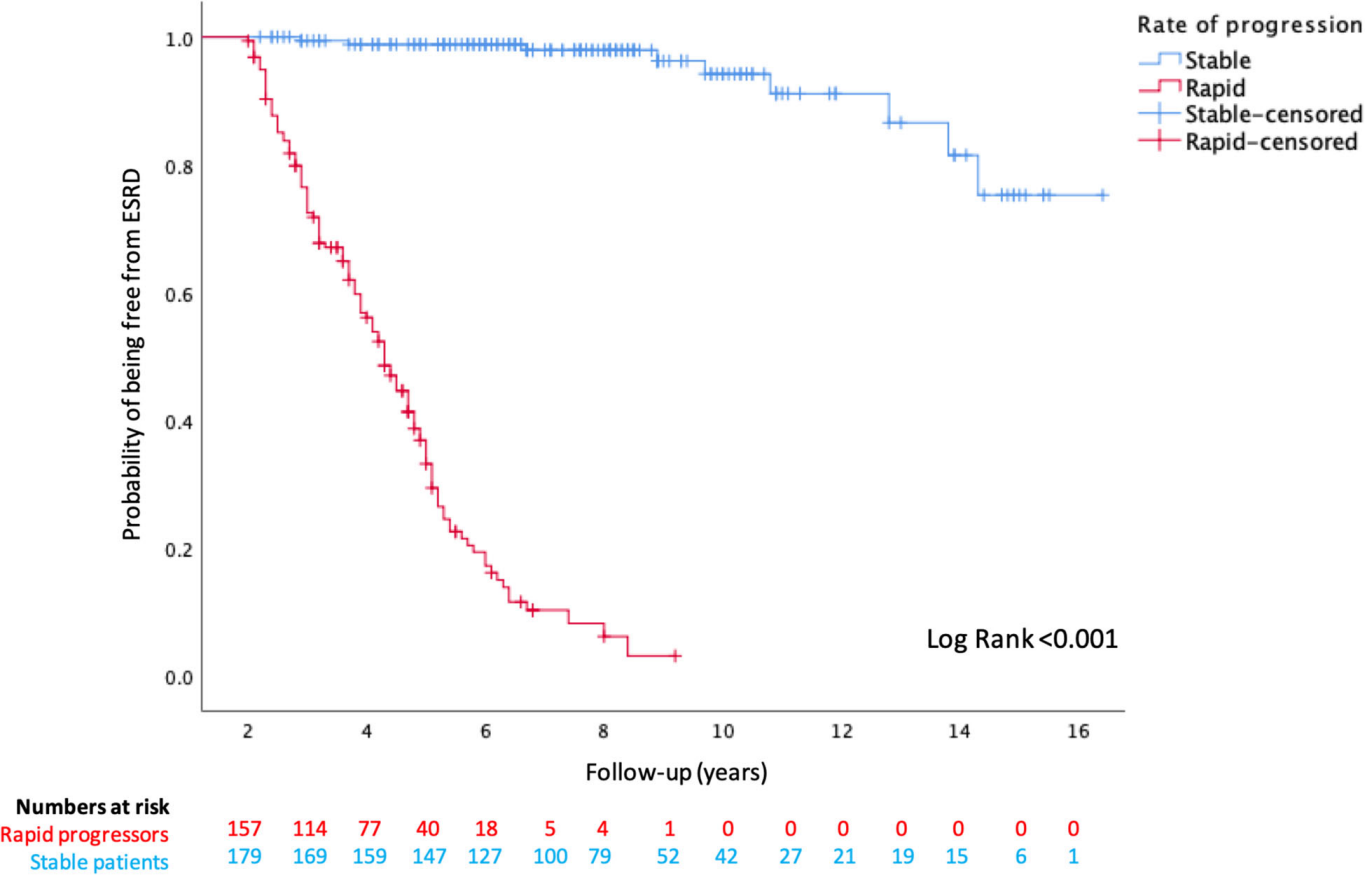
Kaplan Meier curve for probability of survival from ESRD

**Fig. 4:**
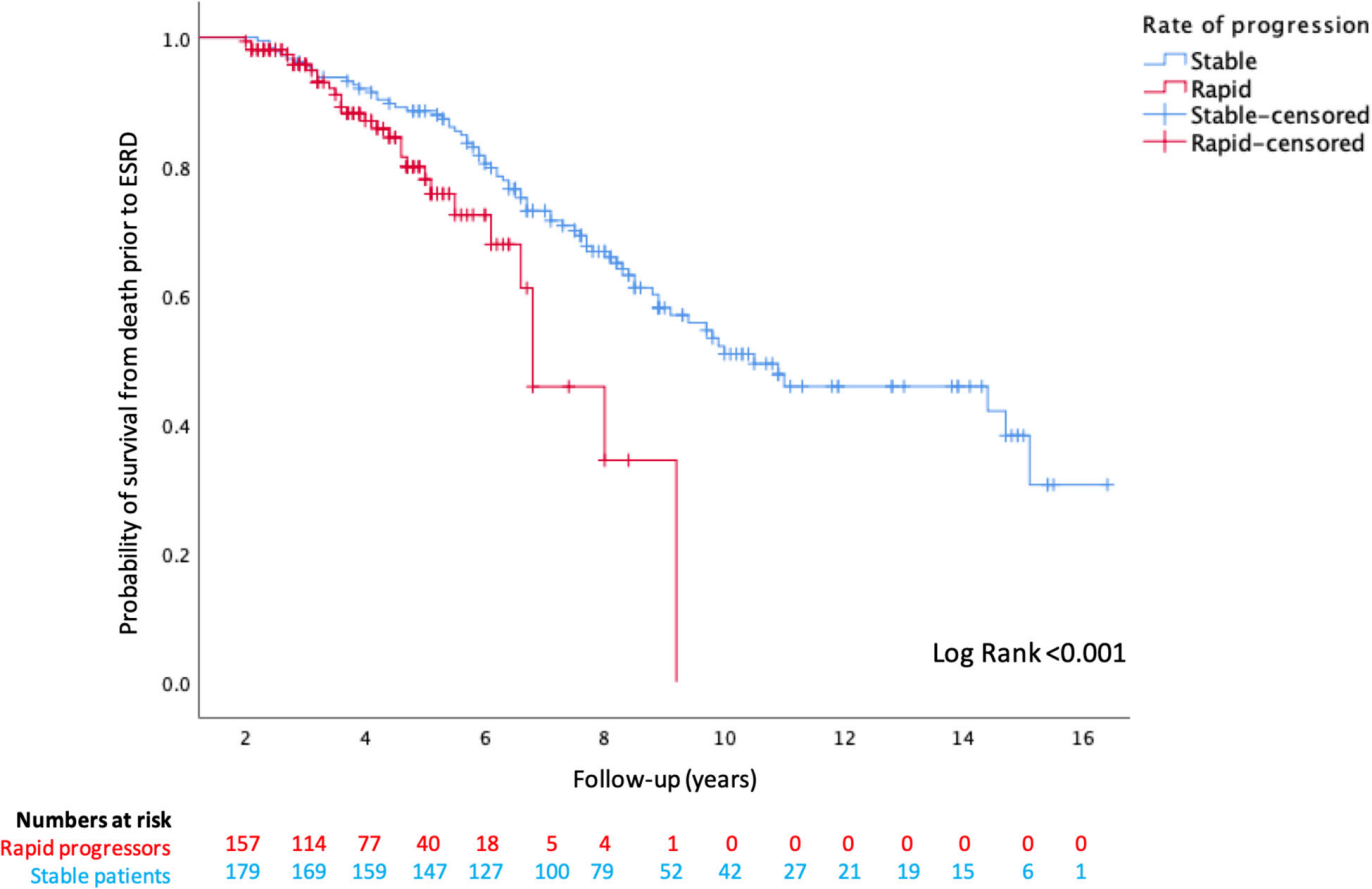
Kaplan Meier curve for probability of survival from death prior to ESRD

**Fig. 5:**
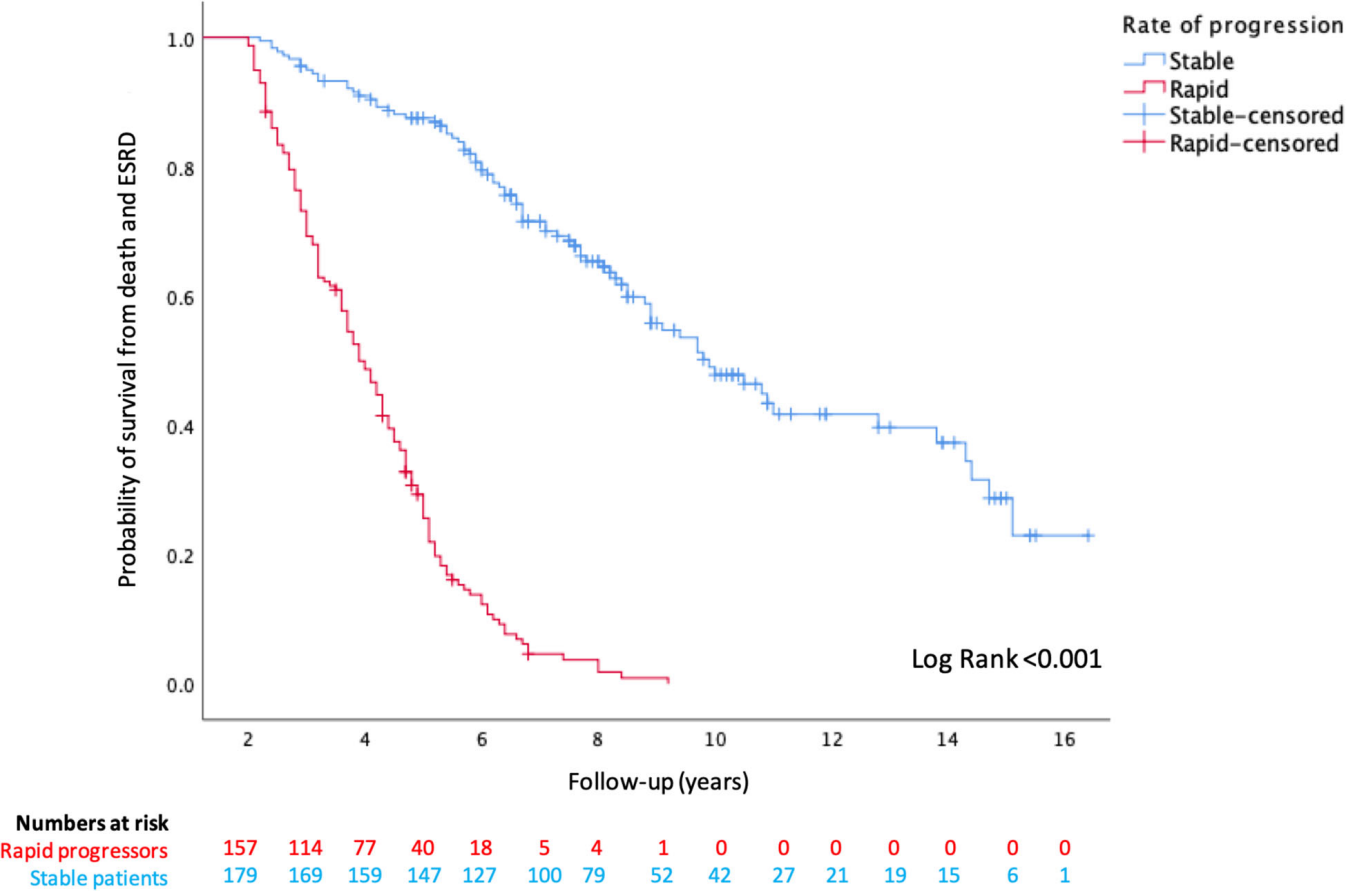
Kaplan Meier curve for probability of survival from ESRD or death prior to ESRD

Kaplan-Meier analysis revealed significantly worse outcomes were faced by rapid progressors, compared with stable patients, for reaching ESRD (censored at death) or mortality prior to ESRD (Figs. 3 and 4), and this is further illustrated in Fig. 5 for the combined endpoint of ESRD and mortality prior to ESRD (censored at the last clinic visit, until 31st December 2019). Over the first 5 years of follow-up, rapid progressors reached ESRD at an average rate of 34 per 100 patients per year compared with 0.2 stable patients per 100 per year. Rapid progressors also faced higher rates of mortality over this time period at a rate of 10 per 100 patients per year, compared with 6 per 100 per year amongst stable patients.

## Discussion

This study highlights several risk factors predictive of rapid linear progression, which are uniquely expressed in different renal diseases. We also highlight distinct clinical factors associated with mortality prior to ESRD in rapid progressors compared with stable patients.

Interventions targeting modifiable factors should be prioritised, especially in rapid progressors, given the significant burden of adverse outcomes experienced by this patient cohort.

### Predictive factors associated with progression

Studies have shown that younger age [17], dyslipidaemia [18], lower albumin [19], lower Hb [20] and proteinuria [21] are associated with CKD progression and all these factors were predictive of patients having rapid linear progression in our analysis. The observation of younger patients at more at risk of progression may be due to the underlying age differences in CKD aetiology [22]. Indeed, in our cohort, a third of the rapid progressors were patients with ADPKD, in whom the median age was 51 (44.8–56.5) years [data not shown], compared to 68.4 (58.8–76.5) years in the stable cohort as a whole.

Of note, we also found female gender to have a positive association with rapid linear progression. Studies that explored gender differences in CKD have found conflicting results: some found male sex confers more risk [23, 24] whereas other studies suggest the opposite [25, 26]. The exact reason for why sex differences exist in patients with CKD is not clearly understood and remains an area for further research.

We also interestingly found that higher DBP was more important than SBP in predicting rapid progression. Although historic studies have highlighted a role of DBP in progression, more recent ones have focussed on the importance of SBP alone [17], or of both SBP and DBP [27], with respect to renal outcomes. We did find higher SBP was associated with rapid progression in the univariate analysis (Additional file 1: Table S3), but it was not significant after adjustment of other covariates. Further work may be required to better understand the clinical implications of DBP in those with advanced CKD, an issue recently identified by the renal community warranting further review [28].

CKD aetiology is important in predicting future progression and our study highlights the well-known association of ADPKD being most commonly linked with rapid linear progression [29] as a consequence of the progressive nature of cyst enlargement and destruction of healthy renal architecture. The higher proportion of stable patients with renovascular disease or obstructive nephropathy in our study is likely reflective of successful treatment interventions that remove ongoing renal injury in these conditions, minimising the risk of developing tubular atrophy and interstitial fibrosis and thus improving long-term renal outcomes.

What is perhaps less well understood is the interplay of factors in the pathogenesis of rapid linear progression in other primary renal disease states. This is shown in the differential impact of exposures on three renal conditions (Table 3). For instance, rapid progressors with diabetic nephropathy were more likely to be anaemic and have A3 proteinuria, whereas rapidly progressing patients diagnosed with glomerulonephritis were more likely to have lower albumin and severe proteinuria, which is indicative of active disease and perhaps inflammation driving renal decline. Higher BMI was also associated with rapid progression in those with glomerulonephritis, but this is likely confounded by patients who were taking immunosuppressive agents such as steroids which can raise BMI.

### Predictive factors associated with mortality

There was an unsurprising representation of cardiovascular risk factors such as older age, male gender, smoking, PVD, HF and A3 proteinuria associated with mortality in both patient groups. However, these factors impacted the two patient groups in different ways. For instance, rapid progressors who had suffered a prior MI were less likely to survive, whereas there was a significant risk of mortality amongst stable patients who had suffered PVD or HF. Whether these differences are directly attributable to pathophysiological processes underlying different rates of progression requires further exploration. A3 proteinuria did not impact mortality in rapid progressors but was important for those who had stable disease. This may due to the potentially greater role severe proteinuria plays on the competing risk of ESRD in rapid progressors. Notably, use of ACEi/ARB was found to reduce the mortality risk in rapid progressors specifically. Although the beneficial effect of ACEi/ARB on mortality at different CKD stages has been highlighted in prior studies [30, 31], we show this benefit extends to those with a defined rate of rapid CKD progression. Potential protective mechanisms include favourable haemodynamic changes [32] on the cardiovascular system but also anti-inflammatory effects of renin-angiotensin-aldosterone blockade [33], which may be of particular relevance in the inflammatory milieu of rapid CKD progression.

### Clinical implications

There are several clinical implications of our findings. Firstly, there is a pressing need for accurate risk stratification that aids prognostication of adverse clinical outcomes in patients with CKD. Developing risk prediction calculators that take account of CKD aetiology or the rate of prior eGFR change, both of which are important determinants that influence future eGFR trajectory [34], would be desirable.

Secondly, our data clearly demonstrate that those with rapid linear progression are an especially vulnerable group of patients that suffer significantly higher annual rates of ESRD or mortality compared to their stable counterparts. Translating this to clinical practice requires assessment of patients’ rate of eGFR decline based on prior blood tests and those progressing rapidly should be offered prompt, vigorous management of modifiable risk factors and closer follow-up monitoring to mitigate future harm.

Finally, we highlight that stable CKD is also not benign. In our cohort, stable patients were older with a higher burden of cardiovascular disease, and although only 5% of patients reached ESRD, 40% of patients died. It underscores previous work showing that older patients are more likely to have stable disease, but that the absolute risk of death in this CKD subgroup remains high, largely as a consequence of cardiovascular disease [35], and this was also borne out in our study. Therefore, an equally important aspect of optimal CKD care, regardless of the rate of progression, requires addressing modifiable cardiovascular risk factors given their association with mortality [21].

### Strengths and limitations

Although several studies have investigated factors predictive of progression, our study has the advantage of providing a closer perspective of those with linear rates of progression using a robust methodological approach to patient selection**.** Each patient had a large number of eGFR measurements taken over a long follow-up period and this helped to precisely characterise patients’ eGFR trajectories. This consequently permitted a robust analysis of patients with different rates of progression, based on their ΔeGFR slope, which was corroborated by visually inspecting each patients’ eGFR-time graphs and confirmed quantitatively by assessing the spread of the 95% CIs of the ΔeGFR in each patient group. Our systematic approach therefore ensured only patients with true linear CKD progression were selected. Our findings also largely support the established literature in describing key determinants of CKD progression and mortality, and in doing so also provides evidence that the phenotypic profile of those with true linear progression is also shared with those with other rates of variable, non-linear progression described in the wider literature.

Our work also has limitations. The analysis was limited to specific ΔeGFR changes to define rapid and stable disease but did not consider the outcomes of other rates of progression, such as those between − 0.5 to -4 ml/min/1.73m^2^/yr or those with larger, positive changes in eGFR over time. This latter group has also been shown to be associated with poor outcomes, perhaps related to changes in muscle mass in patients with chronic illness; or it may represent those whose trajectory is recovering from an episode of acute kidney injury, which is itself has been shown to be an independent risk factor for CKD progression [36]. Changes in muscle mass over time may also be responsible for inaccurate ΔeGFR calculations in older patients, which could not be accounted for in this study. Secondly, our work will be affected by limitations attributed to retrospective observational studies including an inability to confirm causal association or to account for unmeasured confounders. Thirdly, our disease-specific analysis had small numbers of patients and may not be sufficiently powered to define specific predictive associations. We were unable to evaluate risk factors specific to ADPKD for this reason due to there being only 2 patients with stable disease and 52 with rapid progression. Fourthly, it is a single-centre study with a largely Caucasian population and thus the results may not be generalisable to other ethnic patient cohorts in other geographical locations.

## Conclusions

Rapid linear CKD progression represents a confluence of several risk factors, which act heterogeneously depending on the underlying aetiology of CKD. Patients with rapid linear progression are at high risk for adverse clinical outcomes and therefore warrant frequent specialist monitoring. Further refining of current risk prediction tools in CKD will hopefully help optimise care for such high-risk patients.

## Supplementary information

**Additional file 1: Table S1.** Time-by-variable interactions to test proportional hazards assumption for rapid linear progressors. **Table S2.** Time-by-variable interactions to test proportional hazards assumption for stable patients. **Table S3.** Univariate analysis of factors associated with rapid progression. **Table S4.** Baseline characteristics of rapid progressor patients with diabetic nephropathy, glomerulonephritis and hypertensive nephropathy. **Table S5.** Univariate analysis using Cox proportional hazards to evaluate factors associated with mortality prior to ESRD in rapid progressors. **Table S6.** Univariate analysis using Cox proportional hazards to evaluate factors associated with mortality prior to ESRD in stable patients.

## Data Availability

The datasets generated and analysed during the current study are available from the corresponding author on reasonable request.

## Abbreviations

ACEi: Angiotensin converting enzyme inhibitor
ARB: Angiotensin receptor blocker
ADPKD: Autosomal dominant polycystic kidney disease
BMI: Body mass index
CKD: Chronic kidney disease
CI: Confidence interval
ΔeGFR: Delta estimated glomerular filtration rate
DM: Diabetes mellitus
DBP: Diastolic blood pressure
ESRD: End-stage renal disease
eGFR: Estimated glomerular filtration rate
HD: Haemodialysis
Hb: Haemoglobin
HF: Heart failure
HDL: High density lipoprotein
MI: Myocardial infarction
OR: Odds ratio
PD: Peritoneal dialysis
PVD: Peripheral vascular disease
SBP: Systolic blood pressure
SKS: Salford kidney study
uPCR: Urine protein:creatinine ratio
