# Supplementary material for: Predictive factors of rapid linear renal progression and mortality in patients with chronic kidney disease
Source: BMC Nephrol. 2020 Aug 14;21:345. doi: 10.1186/s12882-020-01982-8 (PMC7427893; doi:10.1186/s12882-020-01982-8)
Supplement: Supplementary file 1 — Additional file 1: Table S1. Time-by-variable interactions to test proportional hazards assumption for rapid linear progressors. Table S2. Time-by-variable interactions to test proportional hazards assumption for stable patients. Table S3. Univariate analysis of factors associated with rapid progression. Table S4. Baseline characteristics of rapid progressor patients with diabetic nephropathy, glomerulonephritis and hypertensive nephropathy. Table S5. Univariate analysis using Cox proportional hazards to evaluate factors associated with mortality prior to ESRD in rapid progressors. Table S6. Univariate analysis using Cox proportional hazards to evaluate factors associated with mortality prior to ESRD in stable patients. [file 12882_2020_1982_MOESM1_ESM.docx]

**Table S1.** Time-by-variable interactions to test proportional hazards assumption for rapid linear progressors

|  | **In rapid progressors** | |
| --- | --- | --- |
| **Variable** | **Univariate model**  **HR (95% CI)** | **P-value^1^** |
| Age*time | 0.990 (0.970-1.010) | 0.333 |
| Female*time | 1.404 (0.762-2.586) | 0.277 |
| Systolic blood pressure*time | 1.002 (0.988-1.017) | 0.741 |
| Diastolic blood pressure*time | 1.023 (1.000-1.047) | 0.052 |
| Body mass index*time | 0.972 (0.924-1.023) | 0.271 |
| Hypertension*time | 1.003 (0.000-13657.146) | 1.000 |
| Diabetes mellitus*time | 0.760 (0.450-1.285) | 0.306 |
| Smoking*time | 0.861 (0.508-1.457) | 0.577 |
| Myocardial infarction*time | 2.377 (0.750-7.537) | 0.141 |
| Peripheral vascular disease*time | 0.768 (0.125-4.721) | 0.768 |
| Stroke*time | 1.425 (0.313-6.594) | 0.647 |
| Heart failure*time | 1.045 (0.640-1.705) | 0.861 |
| ACEi/ARB*time | 1.415 (0.834-2.400) | 0.199 |
| Statin*time | 1.401 (0.803-2.445) | 0.235 |
| eGFR*time | 1.022 (0.994-1.050) | 0.124 |
| Bicarbonate*time | 1.022 (0.938-1.114) | 0.619 |
| Calcium*time | 2.439 (0.517-11.496) | 0.260 |
| Phosphate*time | 1.273 (0.323-5.015) | 0.730 |
| Albumin*time | 0.993 (0.953-1.034) | 0.732 |
| Total cholesterol:HDL ratio*time | 1.157 (0.952-1.405) | 0.143 |
| Haemoglobin*time | 1.020 (1.001-1.040) | 0.041 |
| A3 proteinuria*time | 0.583 (0.328-1.035) | 0.066 |

**Abbreviations**: ACEi/ARB (angiotensin converting enzyme inhibitor/angiotensin receptor blocker); eGFR (estimated glomerular filtration rate); HDL (high density lipoprotein).

^1^p-value set at <0.01 for statistical significance based on Bonferroni correction.

**Table S2.** Time-by-variable interactions to test proportional hazards assumption for stable patients

|  | **In stable patients** | |
| --- | --- | --- |
| **Variable** | **Univariate model**  **HR (95% CI)** | **P-value^1^** |
| Age*time | 1.007 (0.997-1.017) | 0.163 |
| Female*time | 1.000 (0.931-1.075) | 0.998 |
| Systolic blood pressure*time | 1.005 (1.001-1.009) | 0.023 |
| Diastolic blood pressure*time | 1.006 (0.997-1.014) | 0.181 |
| Body mass index*time | 0.969 (0.929-1.012) | 0.249 |
| Hypertension*time | 1.776 (1.005-3.139) | 0.048 |
| Diabetes mellitus*time | 0.978 (0.827-1.157) | 0.978 |
| Smoking*time | 0.813 (0.670-0.987) | 0.036 |
| Myocardial infarction*time | 1.001 (0.831-1.205) | 0.992 |
| Peripheral vascular disease*time | 1.020 (0.838-1.243) | 0.841 |
| Stroke*time | 1.042 (0.875-1.241) | 0.643 |
| Heart failure*time | 1.216 (0.873-1.694) | 0.247 |
| ACEi/ARB*time | 0.862 (0.718-1.035) | 0.111 |
| Statin*time | 0.926 (0.774-1.109) | 0.403 |
| eGFR*time | 1.008 (0.999-1.016) | 0.085 |
| Bicarbonate*time | 1.034 (1.007-1.062) | 0.014 |
| Calcium*time | 1.679 (0.807-3.496) | 0.166 |
| Phosphate*time | 0.651 (0.421-1.006) | 0.053 |
| Albumin*time | 1.001 (0.982-1.021) | 0.890 |
| Total cholesterol:HDL ratio*time | 1.017 (0.933-1.108) | 0.706 |
| Haemoglobin*time | 1.002 (0.997-1.007) | 0.495 |
| A3 proteinuria*time | 0.984 (0.785-1.233) | 0.886 |

**Abbreviations**: ACEi/ARB (angiotensin converting enzyme inhibitor/angiotensin receptor blocker); eGFR (estimated glomerular filtration rate); HDL (high density lipoprotein).

^1^p-value set at <0.01 for statistical significance based on Bonferroni correction.

**Table S3.** Univariate analysis of factors associated with rapid progression.

|  | **Rapid progression†** | |
| --- | --- | --- |
| **Variable** | **Univariate model**  **OR (95% CI)** | **P-value** |
| Age (per year) | 0.942 (0.925-0.958) | **<0.001** |
| Female | 0.425 (0.271-0.667) | **<0.001** |
| Systolic blood pressure (per 1mmHg) | 1.018 (1.007-1.030) | **0.002** |
| Diastolic blood pressure (per 1mmHg) | 1.066 (1.044-1.088) | **<0.001** |
| Body mass index (per 1kg/m^2^) | 1.002 (0.965-1.040) | 0.911 |
| Hypertension | 1.648 (0.595-4.564) | 0.337 |
| Diabetes mellitus | 0.591 (0.370-0.943) | **0.027** |
| Smoking | 0.820 (0.521-1.289) | 0.389 |
| Myocardial infarction | 0.651 (0.383-1.106) | 0.112 |
| Peripheral vascular disease | 0.635 (0.279-1.450) | 0.281 |
| Stroke | 1.267 (0.618-2.596) | 0.518 |
| Heart failure | 0.984 (0.520-1.863) | 0.961 |
| ACEi/ARB | 1.287 (0.809-2.046) | 0.287 |
| Statin | 0.769 (0.494-1.195) | 0.243 |
| eGFR (per 1ml/min/1.73m^2^) | 1.057 (1.032-1.082) | **<0.001** |
| Bicarbonate (per 1mmol/L) | 0.966 (0.906-1.031) | 0.297 |
| Calcium (per 0.1mmol/L) | 1.516 (0.318-7.212) | 0.601 |
| Phosphate (per 0.1mmol/L) | 7.896 (2.513-24.807) | **<0.001** |
| Albumin (per 1g/L) | 0.835 (0.784-0.889) | **<0.001** |
| Total cholesterol:HDL ratio | 1.259 (1.069-1.482) | **0.006** |
| Haemoglobin (per 1g/L) | 0.982 (0.968-0.996) | **0.011** |
| A3 proteinuria | 8.250 (4.963-13.712) | **<0.001** |

**†**Binary logistic regression used to derive odds ratio for predictors of rapid progression.

**Abbreviations**: ACEi/ARB (angiotensin converting enzyme inhibitor/angiotensin receptor blocker); eGFR (estimated glomerular filtration rate); HDL (high density lipoprotein).

**Table S4.** Baseline characteristics of rapid progressor patients with diabetic nephropathy, glomerulonephritis and hypertensive nephropathy.

| **Variable** | **Diabetic nephropathy**  **(n=31)** | **Glomerulonephritis**  **(n=26)** | **Hypertensive nephropathy**  **(n=11)** |
| --- | --- | --- | --- |
| Age (years) | 58.2 (51.0-67.1) | 60 (44.9-65.6) | 69.9 (56.5-74.1) |
| Men, *n* (%) | 21 (68) | 14 (54) | 10 (91) |
| Caucasian, *n* (%) | 30 (97) | 26 (100) | 11 (100) |
| Systolic blood pressure (mmHg) | 150 (132-161) | 144 (137-165) | 150 (143-164) |
| Diastolic blood pressure (mmHg) | 76 (70-83) | 81 (73-90) | 80 (71-89) |
| Hypertension, *n* (%) | 31 (100) | 26 (100) | 11 (100) |
| Diabetes, *n* (%) | 31 (100) | 5 (19) | 1 (9) |
| Body mass index (kg/m^2^) | 31.1 (27.3-33.3) | 31.9 (25.5-36.7) | 28.1 (27.1-29.6) |
| Past/current smoking history, *n* (%) | 21 (68) | 18 (69) | 5 (45) |
| Myocardial infarction, *n* (%) | 4 (13) | 0 (0) | 0 (0) |
| Peripheral vascular disease, *n* (%) | 4 (13) | 0 (0) | 1 (9) |
| Stroke, *n* (%) | 5 (16) | 0 (0) | 2 (18) |
| Heart failure, *n* (%) | 2 (6) | 0 (0) | 1 (9) |
| ACEi/ARB, *n* (%) | 24 (77) | 22 (85) | 7 (64) |
| Statin, *n* (%) | 27 (87) | 18 (69) | 7 (64) |
| Years follow-up | 3.6 (3.0-4.4) | 4.2 (2.5-5.4) | 4.2 (3.1-4.7) |
| **Laboratory results** |  |  |  |
| Creatinine (umol/L) | 169 (154-195) | 157 (124-171) | 189 (153-200) |
| eGFR-EPI (ml/min/1.73m^2^) | 34 (27-40) | 39 (34-42) | 33 (28-39) |
| eGFR measurements, *n* | 26 (15-38) | 26 (18-48) | 29 (23-41) |
| ΔGFR (±ml/min/1.73m^2^/yr) | -5.656 (-6.571 to -4.714) | -6.474 (-8.856 to -5.708) | -5.543 (-6.727 to -5.127) |
| Bicarbonate (mmol/L) | 22.6 (21.5-24.7) | 21.9 (20.1-25.6) | 23.2 (20.9-24.7) |
| Urea (mmol/L) | 13.0 (10.7-15.7) | 11.7 (9.4-15.9) | 13.4 (11.6-14.3) |
| Calcium (mmol/L) | 2.31 (2.22-2.39) | 2.28 (2.21-2.39) | 2.32 (2.28-2.38) |
| Phosphate (mmol/L) | 1.19 (1.04-1.36) | 1.17 (1.07-1.31) | 1.05 (0.91-1.13) |
| Alkaline phosphatase (mmol/L) | 94 (79.5-110) | 68.5 (58.0-93.8) | 69 (60-84) |
| Albumin (g/L) | 39 (37-42) | 39 (35-42) | 41 (39-44) |
| Total cholesterol:HDL ratio | 3.52 (2.74-4.92) | 4.04 (3.16-4.46) | 2.87 (2.66-4.12) |
| C-reactive protein (mg/L) | 3.1 (1.4-7.3) | 2.7 (1.2-5.1) | 2.8 (2.0-8.0) |
| Haemoglobin (g/L) | 118 (105-125) | 129 (110-137) | 123 (115-127) |
| Urine protein:creatinine ratio (g/mol) | 269 (107-446) | 270 (153-490) | 130 (67-186) |
| - A1 proteinuria (<15g/mol) | 0 (0) | 0 (0) | 1 (9) |
| - A2 proteinuria (15-50g/mol) | 4 (13) | 2 (8) | 1 (9) |
| - A3 proteinuria (>50g/mol) | 27 (87) | 24 (92) | 9 (82) |

Continuous data presented as median (interquartile range) and categorial data as numbers (percentages).

**Abbreviations**: ACEi/ARB (angiotensin converting enzyme inhibitor/angiotensin receptor blocker); eGFR (estimated glomerular filtration rate); HDL (high density lipoprotein).

**Table S5**. Univariate analysis using Cox proportional hazards to evaluate factors associated with mortality prior to ESRD in rapid progressors.

|  | **In rapid progressors** | |
| --- | --- | --- |
| **Variable** | **Univariate model**  **OR (95% CI)** | **P-value** |
| Age (per year) | 1.094 (1.060-1.128) | **<0.001** |
| Female | 1.075 (0.505-2.286) | 0.852 |
| Systolic blood pressure (per 1mmHg) | 1.007 (0.988-1.027) | 0.480 |
| Diastolic blood pressure (per 1mmHg) | 0.958 (0.930-0.986) | **0.003** |
| Body mass index (per 1kg/m^2^) | 0.964 (0.896-1.036) | 0.318 |
| Hypertension | 21.635 (0.004-127098.803) | 0.487 |
| Diabetes mellitus | 2.344 (1.105-4.969) | **0.026** |
| Smoking | 1.289 (0.567-2.930) | 0.544 |
| Myocardial infarction | 2.995 (1.716-5.226) | **<0.001** |
| Peripheral vascular disease | 7.923 (2.535-24.758) | **<0.001** |
| Stroke | 1.627 (0.539-4.915) | 0.388 |
| Heart failure | 1.628 (0.847-3.127) | 0.144 |
| ACEi/ARB | 0.417 (0.193-0.903) | **0.026** |
| Statin | 2.439 (1.022-5.816) | **0.044** |
| eGFR (per 1ml/min/1.73m^2^) | 0.932 (0.890-0.975) | **0.003** |
| Bicarbonate (per 1mmol/L) | 0.919 (0.810-1.042) | 0.189 |
| Calcium (per 0.1mmol/L) | 1.450 (0.100-20.985) | 0.785 |
| Phosphate (per 0.1mmol/L) | 0.652 (0.093-4.594) | 0.668 |
| Albumin (per 1g/L) | 0.955 (0.899-1.013) | 0.128 |
| Total cholesterol:HDL ratio | 1.060 (0.813-1.381) | 0.668 |
| Haemoglobin (per 1g/L) | 0.963 (0.939-0.987) | **0.003** |
| A3 proteinuria | 1.709 (0.751-3.892) | 0.202 |

**Abbreviations**: ACEi/ARB (angiotensin converting enzyme inhibitor/angiotensin receptor blocker); eGFR (estimated glomerular filtration rate); HDL (high density lipoprotein).

**Table S6.** Univariate analysis using Cox proportional hazards to evaluate factors associated with mortality in stable patients.

|  | **In stable patients** | |
| --- | --- | --- |
| **Variable** | **Univariate model**  **OR (95% CI)** | **P-value** |
| Age (per year) | 1.086 (1.058-1.115) | **<0.001** |
| Female | 1.077 (0.643-1.803) | 0.779 |
| Systolic blood pressure (per 1mmHg) | 1.001 (0.989-1.012) | 0.914 |
| Diastolic blood pressure (per 1mmHg) | 0.970 (0.949-0.992) | **0.007** |
| Body mass index (per 1kg/m^2^) | 0.969 (0.929-1.012) | 0.158 |
| Hypertension | 1.082 (0.394-2.969) | 0.879 |
| Diabetes mellitus | 1.838 (1.158-2.916) | **0.010** |
| Smoking | 1.555 (0.913-2.649) | 0.104 |
| Myocardial infarction | 1.411 (0.899-2.213) | 0.134 |
| Peripheral vascular disease | 1.696 (1.037-2.776) | **0.035** |
| Stroke | 1.258 (0.724-2.186) | 0.416 |
| Heart failure | 2.807 (1.753-4.493) | **<0.001** |
| ACEi/ARB | 0.810 (0.502-1.306) | 0.387 |
| Statin | 1.518 (0.907-2.542) | 0.113 |
| eGFR (per 1ml/min/1.73m^2^) | 0.980 (0.957-1.005) | 0.111 |
| Bicarbonate (per 1mmol/L) | 1.055 (0.986-1.128) | 0.119 |
| Calcium (per 0.1mmol/L) | 1.106 (0.165-6.245) | 0.986 |
| Phosphate (per 0.1mmol/L) | 3.142 (0.939-10.511) | 0.063 |
| Albumin (per 1g/L) | 0.887 (0.837-0.939) | **<0.001** |
| Total cholesterol:HDL ratio | 0.959 (0.783-1.174) | 0.685 |
| Haemoglobin (per 1g/L) | 0.965 (0.950-0.981) | **<0.001** |
| A3 proteinuria | 1.258 (0.676-2.342) | 0.469 |

**Abbreviations**: ACEi/ARB (angiotensin converting enzyme inhibitor/angiotensin receptor blocker); eGFR (estimated glomerular filtration rate); HDL (high density lipoprotein).
